# Predictive values of the modified Mallampati test, upper lip bite test, thyromental distance and ratio of height to thyromental distance to predict difficult laryngoscopy in pediatric elective surgical patients 5–12 years old at selected Addis Ababa governmental hospitals, Ethiopia: a multicenter cross-sectional study

**DOI:** 10.1186/s12871-022-01901-4

**Published:** 2022-11-28

**Authors:** Mulualem Sitot, Wubayehu Amare, Adugna Aregawi

**Affiliations:** Department of Anesthesia, College of Health Sciences, School of Medicine, Addis Ababa University, Addis Ababa, Ethiopia

**Keywords:** Predictive value, Modified Mallampati test, Thyromental distance, Ratio of Height to TMD, Upper lip bite test, Difficult laryngoscopy, Addis Ababa, Ethiopia

## Abstract

**Background:**

Maintaining patent airways is vital in pediatric anesthetic management. Failure to manage and anticipate difficult laryngoscopy (DL) preoperatively is the leading cause of morbidity and mortality. Data on the predictive values of screening parameters in predicting DL are limited in children. Therefore, this study aimed to assess the predictive value of the modified Mallampati test (MMT), upper lip bite test (ULBT), thyromental distance (TMD), and ratio of height to thyromental distance (RHTMD) in predicting DL in children aged 5–12 years at selected Addis Ababa governmental hospitals in Ethiopia.

**Methods:**

A multicenter cross-sectional study was conducted on 141 elective pediatric surgical patients aged 5 to 12 years selected using a systematic random sampling technique at three governmental hospitals from December 1, 2021, to April 30, 2022. The collected data were entered and analysed by SPSS version 26. Chi-square and Fisher’s exact tests were used to compare categorical variables. The receiver operating characteristic curve analysis was used to compare the accuracy of MMT, ULBT, TMD, and RHTMD against DL. A *P* value < 0.05 was considered statistically significant.

**Results:**

The magnitude of DL was 15.6%. MMT has the highest sensitivity (86.4%), specificity (91.6%), and negative predictive value (NPV) (97.3%) compared to other tests. The ULBT also has a high sensitivity (72.7%) and specificity (84%) with comparable diagnostic accuracy (90.8%) with the MMT (*P* < 0.05). The sensitivity, specificity, positive predictive value (PPV), NPV, and accuracy of TMD were 63.6%, 95.8%, 73.7%, 93.4%, and 82.2%, respectively. The RHTMD has the lowest specificity (63.6%), PPV (22.5%), NPV (91.4%), and accuracy (56.7%) in predicting DL.

**Conclusion:**

The MMT and ULBT are good screening tests, followed by the TMD in predicting DL, while the RHTMD was the least accurate predictor. Because no single test has 100% predictive value, a combination of screening tests is advised in pediatrics for predicting DL.

## Background

One of the vital areas of pediatric anesthetic management is maintaining patent airways during anesthesia and surgery. Inability to manage the airway during anesthesia is the leading cause of injury and fatality. Difficult laryngoscopy (DL) is commonly related to difficult intubation under general anesthesia, resulting in intubation failure and potentially leading to fatal consequences [1].

An anesthetist's daily activities of concern are preoperative airway assessment to identify persons at risk of DL or intubation, anticipating it preoperatively and preparedness to manage it. It is necessary to be able to spot people who are at risk of DL or tracheal intubation, particularly in patients who appear to have apparently normal airways [1–3] because unexpected airway difficulties might be distressing where enough qualified staff and equipment are not readily available [4, 5].

Among all anesthesia-related deaths in pediatrics, 13% are related to difficulties during laryngoscopy and intubation [6]. The prevalence of DL ranges from 1% to 20.8% in pediatrics [7–13].

In clinical anesthesia, there are many preoperative screening tests for predicting DL and difficult tracheal intubation [4, 5, 14–19]. However, the diagnostic accuracy is based on adult patients’ findings and varies because of differences in patient ethnicity and physical characteristics [14–17], which requires investigation in pediatrics. Because of the variations in pediatric anatomy and physiology, high vulnerability to respiratory-related adverse events (43% of all adverse events), and limited availability of airway equipment compared to adults, children are at high risk of DL and intubation [4, 5, 18, 19]. Therefore, predicting DL in pediatric patients is important.

Few studies with no public agreement have examined the clinical predictors of laryngoscopy difficulties in children [7, 10, 12, 13, 15], and there are no available data on the study area. Therefore, this study aimed to assess the predictive value of MMT, ULBT, TMD and RHTMD, which are simple to perform and easy to understand, to predict DL in pediatric patients aged 5–12 years with no obvious difficult airway signs at selected Addis Ababa governmental hospitals in Ethiopia. This research will provide information on the predictive values of these screening tests in pediatrics, which will help to detect problematic laryngoscopy during preoperative airway assessment in children and to plan accordingly.

## Methods

### Study area, study design and period

This multicenter hospital-based cross-sectional study was conducted in three governmental hospitals of Addis Ababa, Ethiopia: Tikur Anbessa Specialized Hospital (TASH), Yekatit 12 Hospital Medical College and Menelik II Specialized Hospital. They were selected purposely because the majority of pediatric surgical procedures are performed in these hospitals. The study was conducted from December 1, 2021, to April 30, 2022.

### Population, eligibility criteria, and variables

The source population was all pediatric elective surgical patients aged 5–12 years who underwent surgery under general anesthesia with endotracheal intubation at selected hospitals. The study population was pediatric patients aged 5–12 years who underwent elective surgery under general anesthesia with endotracheal intubation in the selected hospitals who met the inclusion criteria and selected for the sample during the study period.

ASA I and II pediatrics 5–12 years scheduled for elective surgery under general anesthesia with endotracheal intubation during the study period were included. Pediatric patients 5–12 years of age with congenital upper airway malformation, swelling in the neck region, oral mass, restrictive mouth opening due to pathological condition, maxillofacial trauma, protruded teeth, temporomandibular joint ankylosis, burn contracture on the neck, limitation of cervical mobility, and uncooperative pediatric patients were excluded. The dependent variables of this study were difficult laryngoscopy (YES/No) and the predictive values of the four airway parameters in predicting DL, and the independent variables were MMT, TMD, ULBT, and RHTMD.

This study has been registered on the Research Registry and has received a unique identification number (UIN) “researchregistry8122’’ which can be accessed through https://www.researchregistry.com/browse-the-registry#home/. This study is reported according to the Strengthening the Reporting of Observational Studies in Epidemiology (STROBE) criteria [20].

### Sample size and sampling technique

The sample size was determined using a single population proportion formula, with a prevalence of 0.5 and a margin of error of 5% at the 95% confidence interval.

**n =** $\frac{{{(\mathbf{Z}}_{\frac{{\varvec{\upalpha}}}{2}})}^{2}\mathbf{*}\mathbf{p}(1-\mathbf{p})}{{\mathbf{w}}^{2}}$  = 384 where n = sample size, Z = 1.96, p = 0.5 & w = 0.05. Since the target population who underwent surgery in elective bases under general anesthesia with endotracheal intubation during the past three months in the study hospitals was (N = 185), from log book review, a finite population correction formula was used for sample size adjustment (**nf) = **$\frac{{\varvec{n}}}{(1+\frac{{\varvec{n}}}{{\varvec{N}}})}$**=**128. Then, by adding 10% for the nonresponse rate, a total of 141 elective pediatric surgical patients were included in this study.

**Fig. 1:**
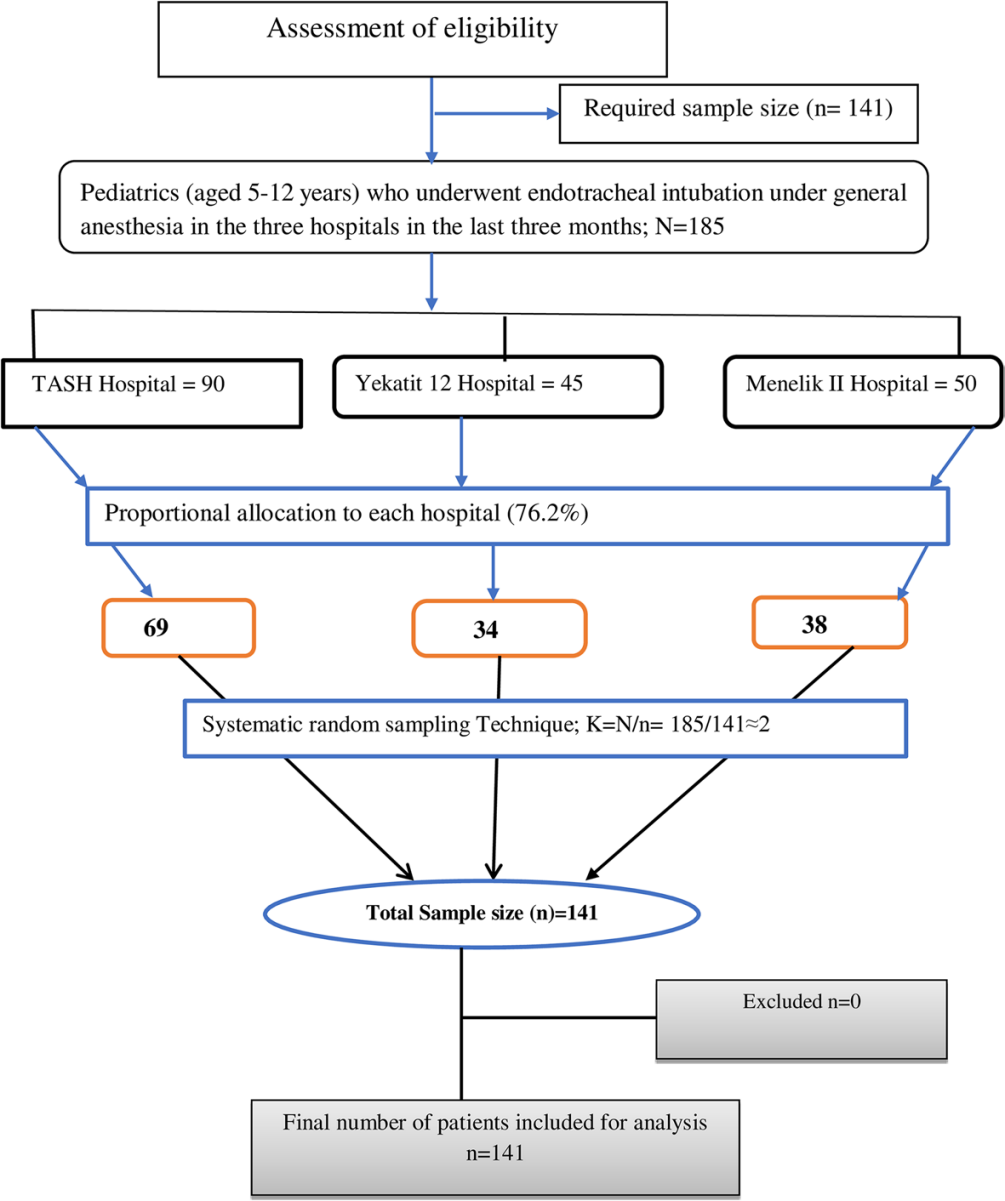
Flow diagram of proportional allocation and sampling technique of the participants. Data collection procedures and quality assurance

Therefore, study subjects for each hospital were selected using proportion allocation by dividing the number of cases during situational analysis in each hospital multiplied by the sample size (*n* = 141) by the total number of patients (*N* = 185). Therefore, 69 at TASH, 34 at Yekatit 12 Hospital Medical College and 38 at Menelik II Specialized Hospital were involved in the sample. Study participants were selected using a systematic random sampling technique using a sampling interval (K): K = N/n; 185/141≈2. Therefore, the first study participant was selected using the lottery method from the daily operation schedule list. Then, every second case was included from each study hospital, using the daily operation elective schedule as a sampling frame (Fig. 1).

Before the study, an ethical approval letter was written from the Institution Review Board (IRB) of Addis Ababa University College of Health Sciences (reference number: Anes 15/2021/2022). Then, a legal letter was submitted to the study hospitals, and ethical clearance was obtained. Data collectors and supervisors were trained on the study objective and the assessment tool for one day before data collection. Before the data collection began, the study participants’ families were informed about the study’s purpose. Then, after obtaining written informed assent, data were collected using a pretested structured questionnaire. Age, sex, and ASA status were collected from the chart. The height (in centimeters) and weight of the children (in kilograms) were measured using a flexible meter and weight balance, respectively. The MMT was graded by visualizing orophargeal structure, while the patient was seated, head in a neutral position, mouth wide open, and tongue protruding maximally. The ULBT was assessed with patient sitting, head in neutral position. and instructed to bite his/her upper lip and graded accordingly. The thyromental distance was measured in the sitting position, with the head completely stretched on the neck and the mouth closed, using a rigid ruler by trained data collectors before the patients entered the operation room. RHTMD was determined by dividing the patient's height by the TMD. After applying standard monitors, preoxygenation was carried out followed by intravenous anesthesia induction and muscle relaxation using Suxamethonium based on milligrams per kilogram of the children. Then, direct laryngoscopy was performed using a Macintosh number 2 blade in 108 cases and a Miller number 2 blade in 33 cases. The patient's Cormack and Lehane laryngoscopic grades were determined by the anesthetist who performed the laryngoscopy by providing diagrammatic representation of the larynx after direct laryngoscopy and completion of intubation. All 141 direct laryngoscopies and endotracheal intubations were performed by anesthetists with a minimum of four years of experience in this study. The supervisors kept an eye on the data gathering process. Each questionnaire was checked for completeness by the principal investigator and the supervisors daily.

### Data analysis and interpretation

Data with complete information were entered and coded into SPSS version 26 (IBM corporate) for analysis. Categorical variables were presented as a number and percentage. Mean and standard deviation were used to express continuous demographic data. Cross tabulation was performed to obtain the numbers of DL in relation to the MMC, ULBT, TMD, and RHTMD. Chi-square and Fisher’s exact tests were used to compare categorical variables, as appropriate. Receiver operating characteristic curve analysis was used to compare the accuracy of MMC, ULBT, TMD, and RHTMD against DL. Cut-off values of TMD and RHTMD were calculated by receiver operating curve analysis. Sensitivity, specificity, positive and negative predictive values with 95% confidence intervals were calculated using cross tabulation to assess the association between the outcome and exposure variables. Statistical significance was defined at a *P* value of less than 0.05.

### Operational definitions

#### Direct laryngoscopy

The procedure performed to visualize the vocal cords using laryngoscope.

#### Difficult laryngoscopy

Inability to visualize the vocal cord during direct laryngoscopy after induction and muscle relaxation (CLG III and/or IV) [21, 22].

#### Cormack and Lehane grade (CLG)

Methods of classifying the degree of visualization of the vocal cord (grade I-IV) during direct laryngoscopy after induction of anesthesia and during direct laryngoscopy [22].

- Grade I: Visualization of the entire laryngeal aperture.
- Grade II: Only the posterior commissure of the laryngeal opening is seen.
- Grade III: Only the epiglottis is visible.
- Grade IV: Any portion of the laryngeal structure is invisible

#### Difficult intubation

Tracheal intubation requires > three attempts or > ten minutes for an experienced anesthetist utilizing direct laryngoscopy [21].

#### American Society of Anesthesiologists (ASA) physical status

A method of classifying patients' physical status, which categorizes patients into six categories based on their systemic wellbeing.

#### Modified Mallampati Test (MMT)

A simple airway assessment widely used to predict difficult laryngoscopy/intubation by viewing oropharyngeal structure, while the patient is seated, head in a neutral position, mouth wide open, and tongue protruding maximally. Based on this test, there are five classes viz: 0, I, II, III and IV [22].

- Class 0: part of the epiglottis is visible
- Class I: hard palate, soft palate, uvula, tonsillar fauces and pillars are visible
- class II: hard palate, soft palate, fauces and uvula-tip are visible
- Class III: the base of the uvula and the soft palate are observable
- Class IV: Only the hard palate is visible, and the soft palate is not visible at all.

MM Class III and IV are considered difficult laryngoscopy.

#### Thyromental distance (TMD)

The distance between the mentum and the thyroid notch measured in centimeters (cm) when the patient's neck is fully extended.

#### Upper lip bite test (ULBT)

Ability of patients to bite the top lip with the lower jaw incisors [18].

- Class I: Lower incisors have the ability to bite the upper lip above the vermilion line
- Class II: Lower incisors have the ability to bite the upper lip below the vermilion line
- Class III: Lower incisors unable to bite the upper lip (difficult laryngoscopy can occur)

#### Ratio of height to thyromental distance (RHTMD)

The patient's height in centimeters (cm) divided by the TMD (in cm).

#### Sensitivity

The conditional likelihood of screening tools properly identifying the presence of difficult laryngoscopy $\frac{true postive}{true positive+false negative}$.

#### Specificity

The chance of screening tools successfully diagnosing laryngoscopy is not difficult $\frac{true negative}{true negative+false postive}$.

#### Positive predictive value

The likelihood of being difficult laryngoscopy for screening tools predicted difficult laryngoscopy $\frac{true postive}{true postive+false postive}$.

#### Negative predictive value

The likelihood of not being difficult laryngoscopy for screening tools predicted not being difficult laryngoscopy $\frac{true negative}{true negative+false negative}$.

#### Limitation of cervical mobility

It is defined as the limitation of cervical extension secondary to cervical injury or fixed atlantooccipital joint.

#### Apparent difficult airway indicator

Any mass in the mouth, large anterior neck mass, short neck, fixed atlantooccipital joint and cervical vertebrae, maxillofacial trauma, protruded teeth, temporomandibular joint ankylosis, burn contracture on the neck.

## Results

### Sociodemographic characteristics

Of the 141 study participants included in this study, more than half (53.2%) were males. The mean age of the participants was 7.73 ± 2.1 years. The mean weight and height of the study participants were 26 ± 7.38 kg and 118.1 ± 13.9 cm, respectively (Table 1).

**Table 1:** Sociodemographic characteristics of study participants in the selected hospitals, Addis Ababa, Ethiopia (*n* = 141)

Variables	Category		
Sex, n (%)	Male	Female	TOTAL
	75 (53.2)	66 (46.8)	141 (100)
Age in years (Mean ± SD)	7.8 ± 2.03	7.65 ± 2.13	7.73 ± 2.1
Weight in kilogram (Mean ± SD)	26 ± 7.6	25.9 ± 7.2	26.00 ± 7.38
Height in centimeter (Mean ± SD)	117.5 ± 13.8	118.8 ± 14.08	118.1 ± 13.9
ASA physical status	Category		(%)
	I	99	70.2%
	II	42	29.8

*n (%)* number (percentage), *SD* Standard deviation, *ASA* American Society of Anesthesiologists’

### Magnitude and predictors of difficult laryngoscopy

In this study, the TMD ranged between 4–7 cm (cm) with a mean and standard deviation of 5.5 ± 0.7 cm, and the RHTMD ranged between 18.33 and 25.60 with a mean and standard deviation of 21.39 ± 1.5. In this study, the overall magnitude of difficult laryngoscopy was 22/141 (15.6%). However, there was no failed or difficult intubation.

The majority of the study participants (*n* = 63, 44.68%) had Cormack and Lehane grade I. There was no Cormack and Lehane grade IV. Similarly, there was no MMC class IV. Out of 112 (79.4%) MMC I-II patients, only 3 (2.13%) had difficult laryngoscopy, however, DL was encountered in 19 (13.47%) patients out of 29 (20.6%) patients who exhibited MMC III. Among 19 (13.5%) patients who had TMD < 5 cm, DL was encountered in 8 patients (*p* < 0.05). However, out of 122 (86.5%) patients with measured TMD > 5 cm, DL was observed in 14 (9.93%) cases. Furthermore, from 106 patients who had ULBT class I-II, DL was observed in 6 cases (Table 2).

**Table 2:** Preoperative airway parameters and their distribution with difficult laryngoscopy among study participants

Predictors	Frequency, n (%)	DL, n (%)
**MMC**		
I	47 (33.3)	0 (0)
II	65 (46.1)	3 (2.13)
III	29 (20.6)	19 (13.47)
IV	0 (0)	0 (0)
**TMD**		
< 5 cm	19 (13.5)	8 (5.67)
≥ 5 cm	122 (86.5)	14 (9.93)
**ULBT class**		
I	40 (28.4)	1 (0.70)
II	66 (46.8)	5 (3.55)
III	34 (24.8)	16 (11.35)
**RHTMD**		
< 21.5	71 (50.4)	16 (11.35)
≥ 21.5	70 (49.6)	6 (4.25)

*n (%)* number (percentage), *DL* Difficult laryngoscopy, *MMC* Modified Mallampati class, *CLG* Cormack and Lehane grade

### Preoperative predictive values of difficult laryngoscopy

A receiver operative characteristic (ROC) curve analysis with the area under the curve (AUC) and the 95% confidence interval (CI) found optimal cut-off points for TMD and RHTMD for the prediction of difficult laryngoscopy of 5 cm and 21.5, respectively.

The accuracy of preoperative tests for difficult laryngoscopy was compared using the receiver operating characteristic (ROC) curve. In this study, the MMT had the highest sensitivity (86.4%) and the best negative predictive value (97.3%) compared to the other three screening tests. The TMD and RHTMD also showed a higher sensitivity (82.2% and 56.7%), respectively. A higher specificity was seen in the TMD (95.8%) and MMT (91.6%). The negative predictive values of all airway parameters were higher (Table 3).

**Table 3:** Sensitivity, specificity, positive predictive value, and negative predictive value for preoperative parameters against difficult laryngoscopy

Parameters	Sn	Sp	PPV	NPV	AUC	SE^a^	95% CI^b^	*P*-value	Accuracy
MMT	86.4%	91.6%	65.6%	97.3%	.917	0.0266	0.865, 0.969	< 0.001	90.8%
RHTMD	72.7%	53.8%	22.5%	91.4%	0.810	0.0453	0.721, 0.899	0.001	56.7%
TMD	63.6%	95.8%	73.7%	93.4%	0.902	0.0285	0.847, 0.958	< 0.001	82.2%
ULBT	72.7%	84.0%	45.7%	94.3%	0.809	0.0472	0.717, 0.902	< 0.001	90.8%

*Sn* Sensitivity, *Sp* Specificity, *PPV* Positive predictive value, *NPV* Negative predictive value, *AUC* Area under the curve, *CI* Confidence interval. *P* values are obtained from Fisher’s exact test

^a^DeLong et al., 1988

^b^AUC ± 1.96 SE

**Fig. 2:**
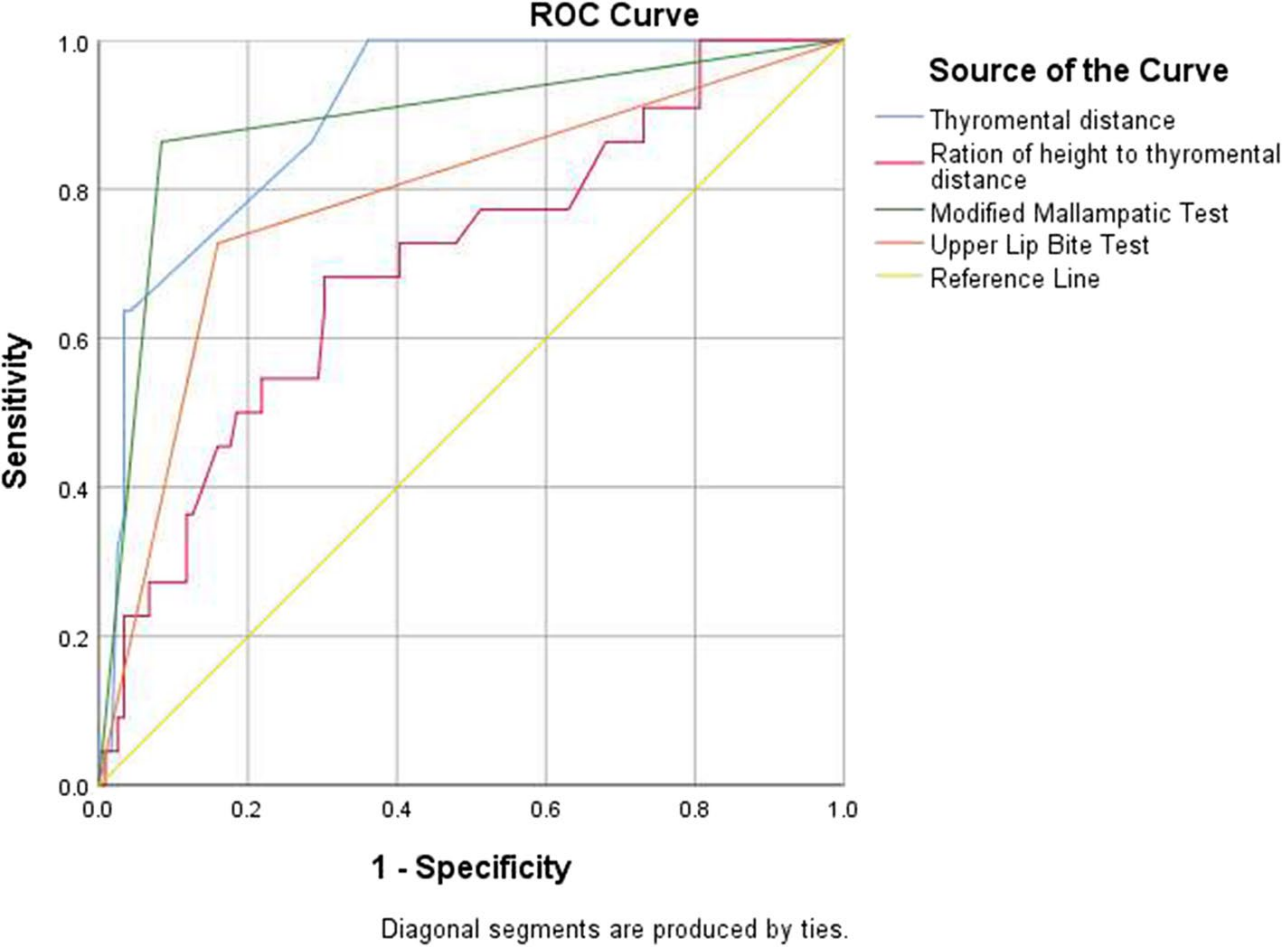
ROC curve for preoperative airway screening tests against difficult laryngoscopy. The pairwise comparison of ROC curves to test the difference between the area under two ROC curves showed a statistically significant difference between ULBT and MMT, RHTMD and MMT, and ULBT and TMD in predicting DL with *P* < 0.05 (Table 4)

The MMT, RHTMD, TMD, and ULBT were all found to be above the reference line (0.5), with areas under the curve of 0.917, 0.810, 0.902, and 0.809, respectively, on the ROC curve (Fig. 2).

**Table 4:** Pairwise comparison of ROC curves for the airway tests

Parameter	Difference between Areas	SE^a^	95% CI	z statistic	*P*-value
ULBT ~ MMT	0.107	0.0529	0.00366—0.211	2.029	0.0424
RHTMD ~ MMT	0.107	0.0491	0.0108—0.203	2.180	0.0293
TMD ~ MMT	0.0143	0.0397	-0.0634—0.0921	0.361	0.7180
ULBT ~ RHTMD	0.000382	0.0616	-0.120 to 0.121	0.00620	0.9951
TMD ~ RHTMD	0.0926	0.0576	-0.0204 to 0.206	1.607	0.1081
ULBT ~ TMD	0.0930	0.0439	0.00706 to 0.179	2.121	0.0339

^a^DeLong et al., 1988

## Discussion

Difficult laryngoscopy is commonly related to difficult intubation under general anesthesia, resulting in failed intubation, which may potentially lead to hypoxia, cardiac arrest and death [1]. The overall magnitude of difficult laryngoscopy was 15.6% among elective pediatric surgical patients (5–12 years), who were old enough to follow instructions, with no apparent difficult airway indicator in our study. The result of our study was higher than other studies that found 1% [7], 1.35% [8], 3% [9], 3.58% [10], 7.2% [11], and 10.4% [12] incidence of DL. In contrast, our result was lower than that of another study [13], which found a 20.8% prevalence of DL. The discrepancies in the magnitude could be due to the use of different types of laryngoscopes which could affect the Cormack and Lehane grading because optimum intubation attempts are associated with alternative laryngoscope blades selection [23]. Furthermore, it could be due to the level of experience of the practitioner which could have a significant association with a difficult laryngoscopy [11, 23, 24], and it could also be due to differences in the patient's ethnicity, anthropology, sample size and definition of difficult laryngoscopy. In our study, there was no Cormack and Lehane grade IV, and there was also no difficult or failed intubation. This could be because direct laryngoscopy and intubation were performed by experienced anesthetists because the skill and experience of the anesthetists can affect the success of laryngoscopy and intubation [4, 21].

In our study, we found 0% and 2.13% difficult laryngoscopy (CLG III) in MMC I, and MMC II patients, respectively. In contrast to our result, Ezri T. et al. [25] found a higher magnitude (3.2%) of CLG III in MMC I patients. K. Murugesan et al. [26] also found a higher magnitude of CLG III; 1.35% and 4.3%, in MMC I, and MMC II patients, respectively. This indicates that the MMC to Cormack and Lehane grade relationship varies from population to population in predicting DL, and predicting easy laryngoscopy in MMT may become difficult.

An ideal preoperative airway screening test should be highly sensitive, specific, and should have high positive predictive value with less negative predictive value to identify patients at risk of difficult laryngoscopy/intubation correctly [27]. In our study, MMT was the most accurate predictor of DL, with fairly high sensitivity (86.4%), specificity (91.6%), moderately high PPV (65.6%), high NPV (97.3%), and high diagnostic accuracy (90.8%) (*p* < 0.001). Thus, the MMT might correctly identify patients who will be at risk of difficult laryngoscopy. On the other hand, MMC being easy preoperatively (MMC I and II) might not rule out the absence of DL.

In line with our study, Sumer S. S et al. [15] found that the MMT has high sensitivity (75%), specificity (92%), and diagnostic accuracy (90%) among the TMD, ULBT and RHTMD. Our result was also in agreement with another study [12] that found the highest diagnostic accuracy (93.6%), moderate to fair sensitivity (76.92%), specificity (95.4%), PPV (66.7%), and NPV (97.3%) for MMT in predicting DL in children.

In our study, the sensitivity, specificity, NPV, and diagnostic accuracy of ULBT were moderate to fairly high (72.7%, 84%, 94.3%, and 90.8%, respectively), while the PPV (45.7%) was poor in predicting DL.

Similar to our study, Mehmet Turan et al. [12] found that the MMT and ULBT are useful in pediatrics aged 5 to 11 years for predicting difficult intubation with moderate to high sensitivity, specificity, and NPV (69.23%-97.32%). The result of our study was also comparable to another study [15] that found a moderate specificity (79.55%) and high NPV (93.33%) for ULBT. In contrast to our study, KILIÇ Y et al. [13] and Khan ZH et al. [18] found that the ULBT had higher specificity and accuracy than the MMT in pediatrics. Eberhart et al. [28] on the other hand found that both ULBT and MMT were poor predictors of DL. The difference may be due to differences in patients’ physical appearance.

Thyromental distance was observed to have moderate sensitivity (63.6%), high specificity (95.8%), NPV (93.4%), PPV (73.7%), and diagnostic accuracy (82.2%) in our study. Unlike adults, there are still no fixed lowest acceptable values for TMD and RHTMD in children, although it will be considerably smaller [29]. According to our study, the cut-off point of TMD was 5 cm in predicting DL by ROC curve analysis. In contrary to our results, a study conducted in India [15] and Turkey [12] found the cut-off value for TMD to be 6.3 cm and 5.5 cm, respectively, in the pediatric population in predicting DL.

In our study, RHTMD is found a poor predictor of DL (low PPV) compared with others. Our study is congruent with a study conducted in India [15] and Turkey [12]. In our study, the cut-off value of RHTMD for predicting difficult laryngoscopy was 21.5 on ROC curve analysis. In contrast to our study, studies conducted in India [15] and Turkey [12] found ideal cut-off values of 18 and 23.5 in predicting DL in pediatric patients, respectively. The possible discrepancies in the cut-off values could be due to anthropological differences among different ethnic groups.

### Strength of the study

This study was conducted in a multicenter setting in homogenous participants, thereby minimizing selection bias, and generalization of the findings is not limited to a single center.

### Limitation of the study

Not using a similar laryngoscopy blade for all cases made it impossible to eliminate interobserver variance bias, which may have influenced the result. Furthermore, the study was conducted on ASA I and II pediatric surgical patients who had normal airways, so we recommended other studies targeting high-risk pediatric populations.

## Conclusion

The magnitude of difficult laryngoscopy was high in apparently normal airway elective pediatric surgical patients aged 5 to 12 years in this study. The MMT is a good test with the highest accuracy for predicting difficult laryngoscopy, followed by the ULBT and TMD. The RHTMD was the least accurate predictor of difficult laryngoscopy in this study. Because no single criterion has 100% predictive value, we recommend using a combination of these four tests to identify difficult laryngoscopy in pediatric patients.

## Data Availability

The datasets generated during the current study are available from the corresponding author on reasonable request.

## Abbreviations

ASA: American Society of Anesthesiologists’ physical status
CLG: Cormack and Lehane grade
DL: Difficult laryngoscopy
MMT: Modified Mallampati test
NPV: Negative predictive value
PPV: Positive predictive value
RHTMD: Ratio of height to thyromental distance
SPSS: Statistical Package for Social Sciences
TMD: Thyromental distance
ULBT: Upper lip bite test
